# Digital Dentistry and Artificial Intelligence: A Systematic Review on Innovations in Diagnosis, Treatment Planning, and Prosthodontics

**DOI:** 10.7759/cureus.104422

**Published:** 2026-02-27

**Authors:** Shruti Karvekar, Vaibhav Anand, Dipti Singh, Vipin Kumar Chaudhary, Sumaiya Fatima, Mohammed Ibrahim Mathar

**Affiliations:** 1 Department of Periodontology, Karnataka Lingayat Education (KLE) Academy of Higher Education and Research (KAHER), Belagavi, IND; 2 Department of Oral and Maxillofacial Surgery, Uttar Pradesh University of Medical Sciences, Saifai, Saifai, IND; 3 Department of Periodontology, Sardar Patel Postgraduate Institute of Dental and Medical Sciences, Lucknow, IND; 4 Department of Computer Science and Engineering, Galgotias College of Engineering and Technology, Greater Noida, IND; 5 Department of Dental Surgery, Kamineni Institute of Dental Sciences, Hyderabad, IND; 6 Department of Prosthetic Dental Sciences, College of Dentistry, Qassim University, Qassim, SAU

**Keywords:** artificial intelligence, diagnostic imaging, digital dentistry, prosthodontics, treatment planning

## Abstract

Digital dentistry has rapidly evolved through the integration of advanced imaging, computer-aided design (CAD) and manufacturing (CAM), and data-driven workflows, creating opportunities for artificial intelligence (AI)-enabled clinical support across multiple dental domains. Although numerous studies have explored AI in isolated diagnostic or restorative applications, a comprehensive synthesis spanning diagnosis, treatment planning, and prosthodontics remains limited, leaving uncertainty regarding the overall scope and maturity of current evidence. This systematic review aimed to consolidate and evaluate published research on AI applications within digital dentistry, with emphasis on data modalities, algorithmic tasks, and reported clinical outcomes. A Preferred Reporting Items for Systematic Reviews and Meta-Analyses (PRISMA)-compliant search was conducted across PubMed/MEDLINE, Scopus, Web of Science, IEEE Xplore, and Google Scholar for studies published between 2015 and 2025. Eligible peer-reviewed studies reporting quantitative outcomes were systematically screened, extracted, and synthesized descriptively. The review identified a predominance of image-based diagnostic applications, particularly for caries detection and anatomical segmentation, with fewer studies addressing treatment planning and prosthodontic workflows. Deep learning models were most frequently employed, and reported outcomes commonly focused on accuracy, segmentation performance, and spatial agreement. The findings indicate that AI is increasingly embedded within digital dental workflows, primarily as a decision support tool rather than a standalone system. This synthesis clarifies current research trends, highlights the relative maturity of diagnostic applications, and underscores the expanding but uneven integration of artificial intelligence across interconnected stages of digital dentistry, providing a consolidated evidence base to inform clinicians and researchers.

## Introduction and background

Digital dentistry has transformed contemporary dental practice by enabling enhanced diagnostic precision, streamlined workflows, and improved predictability of restorative procedures through digital imaging and computer-aided technologies [1]. The widespread adoption of cone-beam computed tomography (CBCT), intraoral scanning, and virtual articulation systems has generated high-dimensional datasets that exceed the interpretive capacity of conventional manual analysis [2]. This increasing data complexity has created a clinical need for advanced computational tools to support accurate and efficient decision-making [3]. Artificial intelligence (AI) has emerged as a methodological framework capable of processing complex dental datasets and extracting clinically meaningful information [4].

Machine learning and deep learning algorithms have been widely applied to dental imaging and surface-based datasets because of their capacity to model complex non-linear patterns [5]. In diagnostic dentistry, AI systems have been evaluated for automated caries detection, periodontal bone loss assessment, periapical pathology identification, and anatomical landmark detection in two- and three-dimensional images [6]. Several studies report diagnostic performance comparable to trained clinicians, particularly in radiographic interpretation tasks [7]. These findings suggest that AI may enhance diagnostic consistency and reduce inter-observer variability when integrated into clinical workflows [8].

Beyond diagnosis, AI applications have extended to treatment planning and prosthodontic rehabilitation [9]. In implant dentistry, AI has been used for implant positioning, assessment of anatomical risk structures, and optimization of digitally guided surgical planning [10]. In prosthodontics, AI has been explored for crown and denture design, occlusal and marginal analysis, and prediction of restorative outcomes within computer-aided design (CAD)/computer-aided manufacturing (CAM) systems [11]. These developments indicate a gradual shift toward automation-assisted prosthodontic processes aimed at improving efficiency and reproducibility [12].

Despite growing research activity, the literature remains fragmented across dental subspecialties and often focuses on isolated AI applications or specific data modalities [13]. Existing evidence frequently evaluates AI within single domains, such as diagnostic imaging or prosthodontics, without examining how these technologies interact across the broader digital dental workflow [14]. Given the interdependence of diagnosis, treatment planning, and restorative execution, this fragmentation limits comprehensive assessment of clinical applicability [7]. The absence of an integrated synthesis complicates evaluation of AI as a cohesive component of modern digital dentistry [2].

Methodological heterogeneity further challenges the interpretation of current evidence. Studies vary considerably in dataset size, reference standards, validation strategies, and outcome reporting [10]. Many investigations rely on retrospective, single-center datasets, raising concerns regarding selection bias and limited generalizability [4]. External validation and prospective clinical testing remain infrequent, although these are essential for real-world implementation [12]. Moreover, most studies emphasize technical accuracy metrics while providing limited evaluation of workflow integration, interpretability, and clinical impact [6].

These limitations underscore the need for a systematic review that integrates AI applications across diagnostic, planning, and prosthodontic domains [1]. A structured synthesis of data modalities, algorithmic approaches, validation strategies, and reported outcomes is required to clarify current capabilities and limitations [9]. Such an approach enables identification of methodological weaknesses that constrain clinical translation and supports development of more robust, clinically aligned AI systems [3,14].

Integrating diagnostic, treatment planning, and prosthodontic domains within a single synthesis allows AI to be evaluated not as isolated technical applications, but as interconnected components of a continuous digital workflow. This perspective enables assessment of clinical readiness and translational potential across the full restorative pathway rather than within individual subspecialties alone.

This systematic review therefore evaluates the current literature on AI applications in digital dentistry, including diagnostic techniques, treatment planning protocols, and prosthodontic workflows. It synthesizes research characteristics, data sources, validation strategies, and reported performance outcomes. Validated methodological tools are used to assess study quality and risk of bias to support transparent interpretation. The objective is to clarify the current stage of AI integration in digital dentistry, identify evidence gaps, and outline priorities for safe and effective clinical implementation.

Objectives of the review

This systematic review aims to synthesize and critically evaluate available evidence on AI applications in digital dentistry across diagnostic assessment, treatment planning, and prosthodontic workflows. It seeks to characterize data modalities, algorithmic strategies, validation approaches, and reported performance metrics, while assessing methodological quality and risk of bias. The review further aims to identify limitations and research gaps that may inform responsible clinical translation and integration of AI into contemporary dental practice.

## Review

Methodology

Search Strategy

A thorough search of the literature was conducted in significant electronic databases to identify studies examining the application of artificial intelligence (AI) in digital dentistry. The databases searched were PubMed/MEDLINE, Scopus, Web of Science, IEEE Xplore, and Google Scholar. The search timeframe was explicitly defined as January 2015 to December 2025.

The primary keywords used across databases included the following: “artificial intelligence,” “machine learning,” “deep learning,” “neural networks,” “convolutional neural network,” “digital dentistry,” “dental informatics,” “prosthodontics,” “diagnosis,” “treatment planning,” “radiograph,” “cone-beam computed tomography,” “CBCT,” “intraoral scan,” “computer-aided design,” and “CAD/CAM.” Truncation and wildcard operators were applied where supported to capture term variations.

For each database, a structured and reproducible search strategy was developed and documented, incorporating database-specific syntax, Medical Subject Headings (MeSH) where applicable, and controlled vocabulary terms. In PubMed, MeSH terms included “Artificial Intelligence,” “Machine Learning,” “Dental Informatics,” and “Prosthodontics,” which were combined with free-text terms. A representative PubMed search string was as follows: (“Artificial Intelligence”[MeSH] OR “Machine Learning”[MeSH] OR “Deep Learning” OR “artificial intelligence” OR “machine learning” OR “deep learning”) AND (“Digital Dentistry” OR “Dental Informatics”[MeSH] OR “prosthodontics”[MeSH] OR “diagnosis” OR “treatment planning”) AND (“radiograph” OR “cone-beam computed tomography” OR “CBCT” OR “intraoral scan” OR “computer-aided design” OR “CAD”). Equivalent Boolean logic and truncation operators were adapted for Scopus, Web of Science, and IEEE Xplore according to their indexing systems.

The search was limited to English-language studies involving human subjects. Grey literature searches were conducted through Google Scholar using structured keyword combinations, and the first 200 results sorted by relevance were screened. Reference lists of eligible publications were manually screened to identify additional relevant studies.

A search of the databases yielded a preliminary set of records, and duplicates were removed using reference management software prior to screening. The number of records identified, screened, excluded, and included was documented in accordance with Preferred Reporting Items for Systematic Reviews and Meta-Analyses (PRISMA) guidelines to ensure transparency and reproducibility.

Eligibility Criteria

Inclusion criteria: The inclusion criteria were to include studies that were peer-reviewed and original research studies investigating the application of AI in digital dentistry with quantitative assessment outcomes. Qualified articles covered diagnostic activities, the treatment planning process, or prosthodontic procedures with digital dental data, that is, radiographs, cone-beam computed tomography (CT) images, intraoral scans, or computer-aided design (CAD) files. Experimental and observational designs of the study were considered. Eligibility criteria were predefined and pilot-tested prior to formal screening to minimize selection bias and ensure consistent application across reviewers.

Exclusion criteria: The exclusion criteria were narrative or systematic reviews, editorials, conference abstracts that did not have full-text data, case reports, non-comparative studies, animal or in vitro studies that did not have sufficient outcome data, non-English articles, and articles that did not have adequate outcome data. Research that did not pass the eligibility criteria in the screening process was eliminated and reported.

The relatively small number of included studies (n = 11) reflects the focused scope on quantitatively validated AI applications in clinical digital dentistry, rather than overly restrictive criteria. Sensitivity checks were performed during screening to ensure that potentially relevant studies were not inadvertently omitted.

Screening Process

Title and abstract screening was conducted independently by two reviewers with prior training in systematic review methodology. Each reviewer applied the predefined eligibility criteria to all retrieved records. Studies considered potentially relevant by either reviewer were advanced to full-text assessment to minimize the risk of premature exclusion.

Full-text screening was performed independently by the same two reviewers. Disagreements were resolved through structured discussion aimed at reaching consensus. If consensus could not be achieved, a third senior reviewer independently evaluated the study and made the final decision.

Data Extraction and Analysis

Data extraction was performed independently by two reviewers using a standardized and pilot-tested data extraction form. Extracted variables included study design, clinical application area, population/sample characteristics, type of digital dental data used, AI methodology, validation approach, and outcome measures. Outcomes of interest included diagnostic performance metrics, treatment planning accuracy or deviation measures, prosthodontic design performance indicators, and workflow-related outcomes where available.

Following independent extraction, extracted datasets were compared, and discrepancies were resolved through discussion and cross-checking against the original full-text articles. Persistent disagreements were resolved through consultation with the third senior reviewer to ensure methodological consistency and accuracy.

The heterogeneity of datasets, AI architectures, validation strategies, and outcome reporting precluded quantitative meta-analysis. Clinical, methodological, and statistical heterogeneity were assessed by comparing study populations, AI model types, validation frameworks, and reported outcome metrics across studies. Due to substantial heterogeneity in outcome definitions and validation procedures, statistical pooling was deemed inappropriate.

Data were synthesized narratively and summarized using structured tables to facilitate comparative analysis. Studies were stratified according to study design (randomized controlled trials, observational clinical studies, retrospective validation studies, and model development studies) to account for differing levels of evidence, and findings were interpreted with hierarchical consideration of study design strength.

Quality Assessment

The methodological quality of included studies was evaluated to determine the strength and reliability of the evidence. Quality assessment focused on clarity of study objectives, appropriateness of study design, adequacy of data sources, validity of outcome measurement, and robustness of validation procedures. Diagnostic accuracy studies were assessed using criteria relevant to imaging-based evaluations, and prediction or treatment planning studies were assessed according to methodological principles applicable to model development and validation.

An evidence grading approach consistent with GRADE principles was applied to evaluate overall certainty of evidence across key outcome domains, considering study limitations, inconsistency, indirectness, imprecision, and publication bias. Certainty of evidence was categorized as high, moderate, low, or very low.

Quality assessments informed the interpretation of findings, although no study was excluded solely on the basis of methodological quality.

Risk of Bias Assessment

Risk of bias was evaluated at the individual study level using validated methodological tools selected according to study design. Randomized controlled trials were assessed using the Cochrane Risk of Bias 2 (RoB 2) tool. Diagnostic accuracy studies were assessed using QUADAS-2, evaluating risk of bias across patient selection, conduct and interpretation of the index test, reference standard, and flow and timing. Non-randomized interventional and observational comparative studies were assessed using the ROBINS-I tool, covering bias due to confounding, participant selection, classification of interventions, deviations from intended interventions, missing data, outcome measurement, and selective reporting. Prediction model development and validation studies were assessed using PROBAST, evaluating bias related to participants, predictors, outcomes, and statistical analysis.

The types of bias evaluated included selection bias related to dataset acquisition, performance bias associated with model training and testing procedures, detection bias related to reference standards and outcome assessment, and reporting bias in results presentation. Particular attention was given to the use of independent test datasets, external validation, transparency of analytical methods, and risk of overfitting. Studies were categorized as having low risk of bias, some concerns, or high risk of bias, depending on the criteria of the respective assessment tool.

Risk of bias assessments were conducted independently by two reviewers. Disagreements were resolved through discussion and consensus, and unresolved cases were adjudicated by a third senior reviewer. Risk of bias judgments were incorporated into the interpretation of findings, with greater emphasis placed on studies demonstrating external validation, prospective design, multi-center datasets, and low risk of bias ratings.

Quality Assessment and Evidence Certainty

The methodological quality of included studies was evaluated to determine the strength and reliability of the evidence. Quality assessment focused on clarity of study objectives, appropriateness of study design, adequacy of data sources, validity of outcome measurement, and robustness of validation procedures.

In addition to study-level quality assessment, certainty of evidence across key outcome domains was evaluated using an evidence grading approach aligned with GRADE principles. Certainty was judged by considering risk of bias, inconsistency, indirectness, imprecision, and publication bias, and was classified as high, moderate, low, or very low. This approach was used to strengthen the interpretation of result reliability and to avoid overinterpretation of findings derived from weaker evidence.

Study Generalizability

Most included studies were retrospective and conducted in single-center settings, which limits external validity and reduces generalizability across diverse clinical environments. This limitation was explicitly considered in evidence interpretation, particularly for AI models trained on narrowly defined datasets or single-device imaging sources. Findings were therefore interpreted as preliminary and context-dependent, highlighting the need for future multi-center prospective validation studies to support clinical translation.

Results

Study Selection

**Figure 1:**
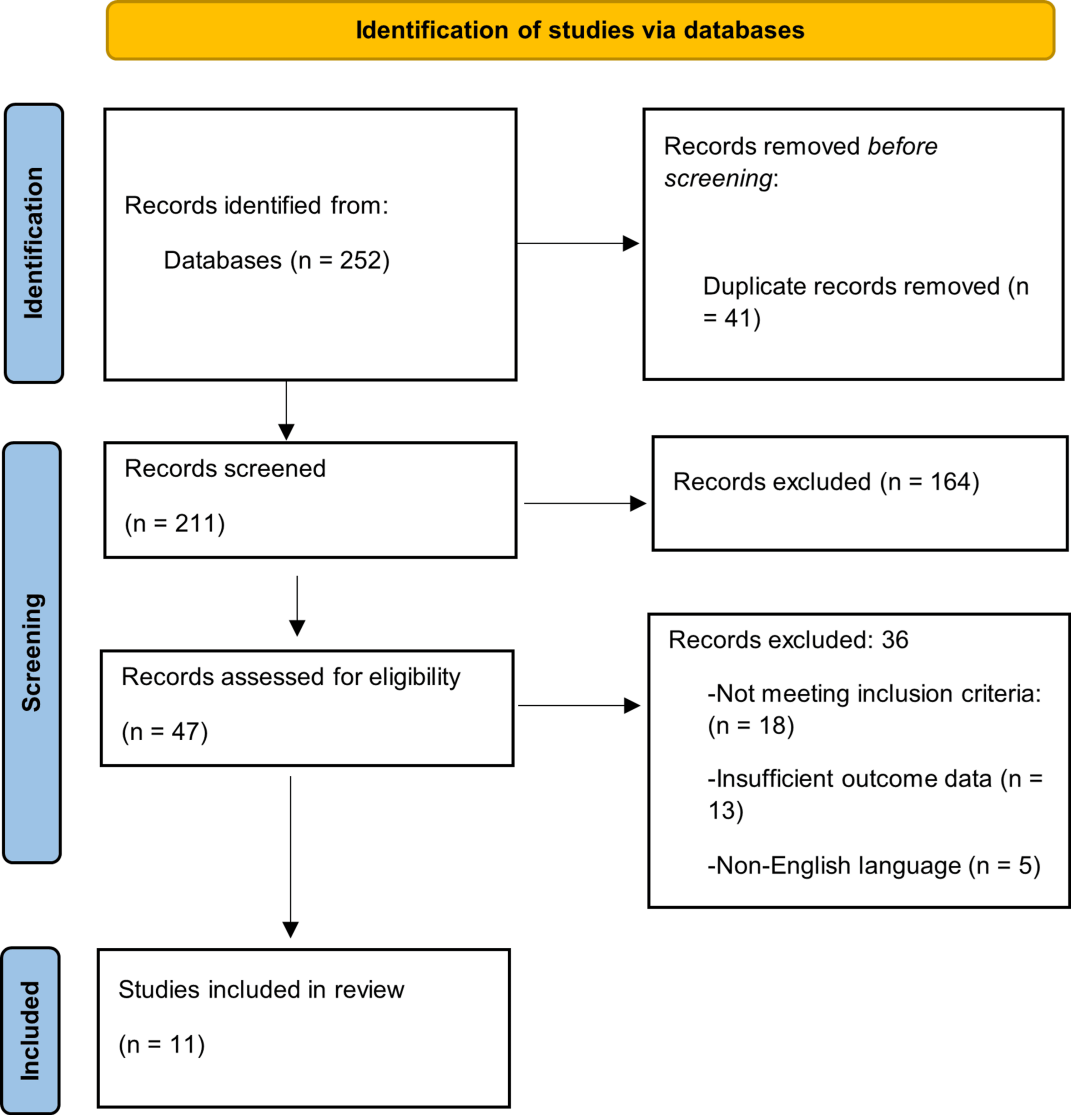
PRISMA flow diagram PRISMA: Preferred Reporting Items for Systematic Reviews and Meta-Analyses (PRISMA)

The computerized search through the database yielded 252 documents whose records were left to be screened by filtering away 41 duplicate records. A total of 164 records were filtered out as a result of title and abstract screening. Then, the eligibility of 47 full-text publications was considered. Among the 36 studies that were eliminated, 13 studies had inadequate data on outcomes. Five studies were published in non-English languages, and 18 studies failed to fulfil the inclusion criteria. The qualitative synthesis included 11 studies. Figure 1 shows the process of selecting the studies through the PRISMA flow diagram.

Characteristics of Included Studies

The findings originated in different geographic regions, both in the academic and clinical settings, as well as in the research settings. Most of the investigations were image-based data, such as panoramic and periapical radiographs, cone-beam (CT) images, and intraoral scans. AI models were primarily built on deep learning models, specifically convolutional neural networks (CNNs). Sample sizes varied from small single-center datasets to some larger multi-image datasets. An overview of study characteristics is displayed in Table 1.

**Table 1:** Key study characteristics and findings of AI applications in digital dentistry AI: artificial intelligence, CBCT: cone-beam computed tomography, CNN: convolutional neural network, RCT: randomized controlled trial, CAD: computer-aided design, CAM: computer-aided manufacturing Studies were grouped by AI task maturity and clinical readiness. Randomized diagnostic trials showed the highest readiness, planning and risk assessment studies demonstrated intermediate readiness, and segmentation and prosthodontic design studies were largely exploratory due to validation-based designs

Study	Clinical application	Study design and population	Data modality	Main outcomes assessed	Key findings	Clinical readiness/task maturity
Mertens et al. [15]	Caries detection	Randomized controlled trial, adult patients	Dental radiographs	Diagnostic accuracy, sensitivity, specificity	AI-assisted caries detection showed higher diagnostic accuracy compared with unaided assessment.	Near-clinical translation (RCT, clinically interpretable endpoints)
Kahya Karaca et al. [16]	Prosthodontic impressions	Clinical comparative study, edentulous patients	Digital and conventional impressions	Accuracy, patient comfort, workflow efficiency	The digital impression approach demonstrated comparable accuracy with improved workflow efficiency.	Clinically promising (comparative clinical study, workflow outcomes)
Ayidh Alqahtani et al. [17]	Tooth segmentation and classification	Validation study, orthodontic patients	CBCT images	Segmentation accuracy, classification performance	Deep learning achieved high accuracy in automated tooth segmentation and classification.	Exploratory, promising (validation study, no clinical workflow testing)
Preda et al. [18]	Maxillofacial structure segmentation	Retrospective validation study, imaging datasets	CBCT images	Dice coefficient, segmentation overlap	Automated segmentation demonstrated high agreement with reference standards.	Exploratory (retrospective segmentation validation)
Tao et al. [19]	Orthodontic implant site planning	Observational study, orthodontic patients	CBCT images	Spatial accuracy, agreement with expert assessment	AI-assisted planning identified suitable implant sites with high agreement to expert evaluation.	Clinically promising (planning decision support, limited by observational design)
Pul et al. [20]	Periapical lesion detection	Randomized controlled trial, clinical cases	Periapical radiographs	Diagnostic accuracy, clinician performance	AI assistance improved the detection of periapical radiolucencies compared with conventional diagnosis.	Near-clinical translation (RCT, clinician performance measured)
Picoli et al. [21]	Nerve injury risk assessment	Within-patient comparative study	CBCT images	Prediction accuracy, risk stratification	AI models accurately assessed the risk of inferior alveolar nerve injury.	Clinically promising (direct clinical risk outcome, limited by design)
Arsiwala-Scheppach et al. [22]	Caries detection support	Randomized controlled trial, dentists	Dental radiographs	Diagnostic accuracy, gaze behavior	AI support altered diagnostic behavior and improved caries detection performance.	Near-clinical translation (RCT, behavioral + accuracy outcomes)
Hegde et al. [23]	Radiographic caries diagnosis	Comparative experimental study	Dental radiographs	Accuracy, sensitivity, specificity	Machine learning models outperformed traditional diagnostic methods.	Clinically promising (comparative, not workflow-embedded)
Cho et al. [24]	Airway segmentation	Validation study, orthodontic patients	CBCT images	Segmentation accuracy	CNN-based segmentation achieved high accuracy across skeletal patterns.	Exploratory (segmentation validation, indirect clinical outcome)
Kong and Kim [25]	Crown prosthesis design	Dataset analysis	Digital crown datasets	Design accuracy, predictive performance	AI-assisted crown design demonstrated consistent prosthetic design outcomes.	Exploratory (technical dataset study, limited clinical validation)

Artificial Intelligence Applications in Diagnosis

AI applications in diagnosis are essentially concerned with radiographic interpretation applications. These included automated recognition plus categorization of dental caries, periodontal bone loss, and periapical radiolucencies, in addition to segmentation of dental and maxillofacial structures. Panoramic radiographs and CT with cone-beam were the most often used imaging tools. Diagnostic accuracy, sensitivity, specificity, area under the receiver operating characteristic curve, and segmentation overlap metrics were reported. Diagnostic performance resulting from the studies is summarized in Table 2.

**Table 2:** Synthesis of findings for AI applications in diagnosis and treatment planning CBCT: cone-beam computed tomography, AI: artificial intelligence

Clinical domain	AI task	Data modality	Outcome metrics reported	Validation approach	Clinical readiness	Reference
Diagnosis	Caries detection	Dental radiographs	Accuracy, sensitivity, specificity	Randomized clinical evaluation	High	[15]
Diagnosis	Tooth segmentation and classification	CBCT images	Segmentation accuracy, classification performance	Internal validation	Low	[17]
Diagnosis	Maxillofacial structure segmentation	CBCT images	Dice coefficient, overlap metrics	Retrospective validation	Low	[18]
Diagnosis	Periapical lesion detection	Periapical radiographs	Diagnostic accuracy	Randomized controlled evaluation	High	[20]
Diagnosis	Radiographic caries detection	Dental radiographs	Accuracy, sensitivity, specificity	Comparative experimental evaluation	Moderate	[23]
Treatment planning	Orthodontic mini-implant site identification	CBCT images	Spatial accuracy, agreement	Observational validation	Moderate	[19]
Treatment planning	Nerve injury risk prediction	CBCT images	Prediction accuracy, risk stratification	Within-patient comparison	Moderate	[21]

Artificial Intelligence Applications in Treatment Planning

**Figure 2:**
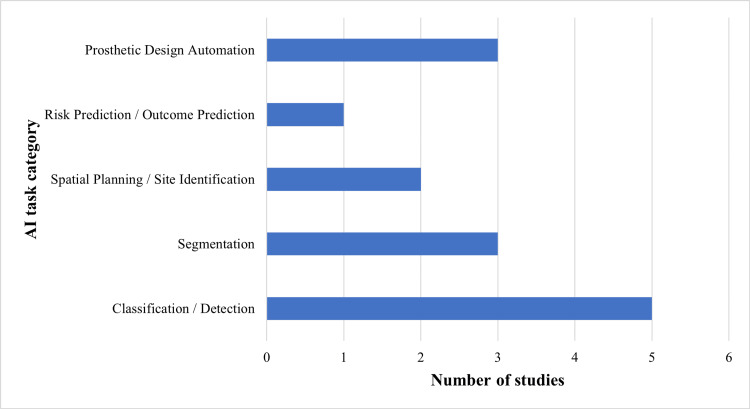
Distribution of AI tasks across clinical applications AI: artificial intelligence

Studies dealing with treatment planning testified to the role of AI to aid implant positioning, evaluation of anatomical limitations, and virtual planning of dental interventions. Cone-beam computed tomography data were very commonly used to get the spatial information for these applications. Reported outcomes were measures of spatial deviation, agreement with reference plans, and planning accuracy, as shown in the distribution of AI tasks in clinical domains in Figure 2.

Artificial Intelligence Applications in Prosthodontics

Prosthodontic applications for AI were aimed at the automation of the design of crowns and dentures, margin detection, occlusal surface analysis, and prediction of restorative results. Digital impressions taken from intraoral scanners and CAD/CAM data sources were the primary source of data. Reported outcomes were design accuracy, prediction error, and agreement to reference standards. The results of prosthodontic are summarized descriptively in Table 3.

**Table 3:** Synthesis of findings for AI applications in prosthodontics CAD: computer-aided design, CAM: computer-aided manufacturing, AI: artificial intelligence

Prosthodontic application	AI task	Data modality	Outcome metrics reported	Clinical context	Clinical readiness	Reference
Digital impressions	Comparison of digital versus conventional impressions	Intraoral scans	Accuracy, workflow efficiency	Edentulous maxilla	Moderate-high	[16]
Crown prosthesis design	Automated prosthetic design	Digital crown datasets	Design accuracy, prediction performance	Fixed prosthodontics	Low	[25]
Prosthodontic restoration	AI-assisted restoration planning	CAD/CAM datasets	Predictive consistency	Prosthodontic rehabilitation	Low	[18]
Prosthodontic workflows	Automated design and planning support	Digital dentistry datasets	Design reliability	Clinical prosthodontics	Low	[11]
Prosthodontic and implant workflows	Integrated AI planning systems	Digital imaging and design data	Workflow optimization	Implant-supported prosthodontics	Moderate	[15]

**Figure 3:**
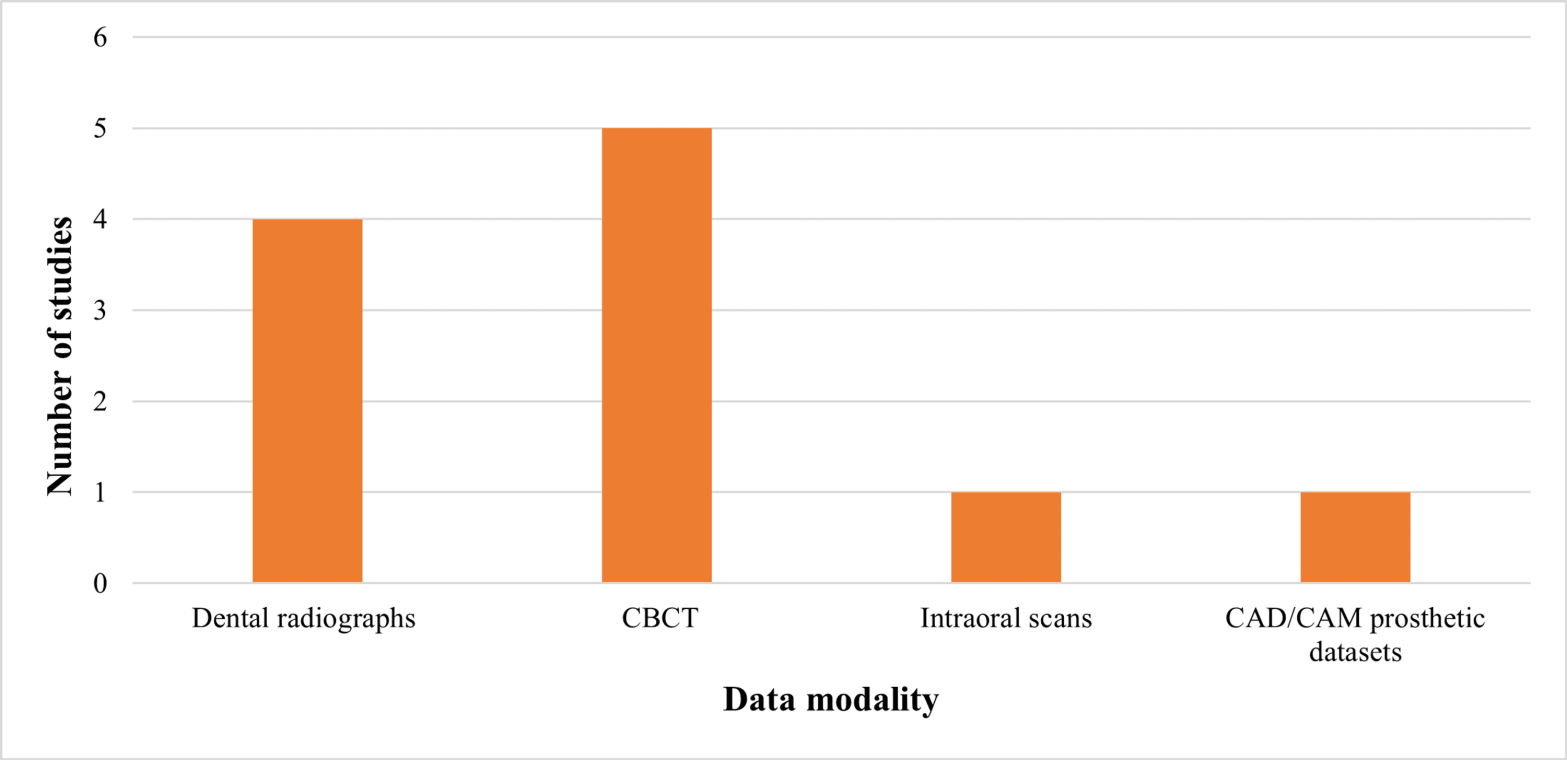
Data modalities used in AI-based digital dentistry applications CBCT: cone-beam computed tomography, CAD: computer-aided design, CAM: computer-aided manufacturing, AI: artificial intelligence

This pattern is demonstrated in the synthesized distribution of data modalities underpinning AI applications, as illustrated in Figure 3.

Outcome Measures Across Studies

Outcome measures were not the same across clinical applications. Diagnostic studies were focused mainly on the classification and detection results, while the treatment planning studies were focused more on the results of spatial accuracy and deviation. Prosthodontic studies reported study outcomes with regard to design precision and predictive performances.

Quality-Related Findings

The included studies showed variability in reporting clarity, characteristics of the datasets, and evaluation rigor. There were some differences in the completeness of outcome reporting and performance measures.

Risk of Bias-Related Findings

Risk of bias-related findings varied among the included studies. Differences were detected in areas related to data selection, outcome assessment, and reporting. Studies were categorized into several levels of risk, and the distribution of these categorizations is given in Table 4.

**Table 4:** Risk of bias assessment of included studies evaluating AI applications in digital dentistry AI: artificial intelligence

Study	Study design	Selection bias	Performance bias	Detection bias	Overall risk of bias
Mertens et al. [15]	Randomized controlled trial	Low	Low	Low	Low
Kahya Karaca et al. [16]	Clinical comparative study	Moderate	Moderate	Low	Moderate
Ayidh Alqahtani et al. [17]	Validation study	Moderate	Low	Low	Moderate
Preda et al. [18]	Retrospective validation study	Moderate	Low	Low	Moderate
Tao et al. [19]	Observational study	Moderate	Moderate	Low	Moderate
Pul et al. [20]	Randomized controlled trial	Low	Low	Low	Low
Picoli et al. [21]	Within-patient comparative study	Low	Moderate	Low	Moderate
Arsiwala-Scheppach et al. [22]	Randomized controlled trial	Low	Low	Moderate	Moderate
Hegde et al. [23]	Comparative experimental study	Moderate	Moderate	Low	Moderate
Cho et al. [24]	Validation study	Moderate	Low	Low	Moderate
Kong and Kim [25]	Scoping review-derived dataset analysis	Moderate	Moderate	Moderate	Moderate

Discussion

This systematic review synthesized evidence regarding the use of AI across diagnostic assessment, treatment planning, and prosthodontic workflows in digital dentistry. The final inclusion of 11 studies from a substantially larger initial pool reflects the strict application of predefined eligibility criteria requiring quantitative outcome assessment, clinical relevance, and sufficient methodological transparency.

The included studies showed a dominance of image-based diagnostic investigations and a relatively limited number of planning and restorative applications. Diagnostic investigations most commonly employed deep learning models trained on radiographic and three-dimensional imaging data, with performance measured in terms of accuracy, sensitivity, and segmentation agreement. Treatment planning studies primarily focused on implant positioning and anatomical risk evaluation using cone-beam CT datasets. Prosthodontic applications targeted digital impressions and automated prosthesis design, with outcomes centered on design accuracy and workflow efficiency.

AI applications in dentistry encompass diverse tasks such as detection, segmentation, spatial planning, and automated design, each using distinct evaluation frameworks and performance metrics. This methodological variability limited direct comparability across studies and precluded quantitative synthesis.

The emphasis on diagnostic applications aligns with established trends in AI research in dental medicine, where radiographic interpretation represents a structured and data-rich use case for algorithm development [26]. Prior syntheses have suggested that structured imaging datasets facilitate the development and evaluation of AI systems with measurable diagnostic performance [27]. The lower representation of treatment planning and prosthodontic studies reflects the greater complexity of these tasks, which require integration of anatomical, functional, and procedural variables [28]. Diagnostic outcomes remain the most frequently reported in the literature, underscoring the central role of imaging-based tasks in digital dentistry research [29].

Deep learning architectures, particularly convolutional neural networks, predominated among included studies, consistent with evidence describing their efficiency in feature extraction from dental imaging data [30]. These architectures are well-suited for processing radiographs and volumetric scans, explaining their widespread use in diagnostic and segmentation tasks [31]. Most studies relied on retrospective validation designs, consistent with earlier reviews of AI applications in dentistry [32].

Previous reviews have identified substantial overlap in diagnostic use cases such as caries detection and anatomical segmentation, indicating convergence of research focus in the field [33]. The present synthesis extends prior work by integrating diagnostic, planning, and prosthodontic applications within a unified framework, enabling a broader perspective on AI integration across digital dental workflows [34]. This integrated perspective illustrates how improvements in diagnostic accuracy may influence downstream planning and restorative procedures [10].

Treatment planning applications were predominantly related to implant dentistry, reflecting the dependence of implant workflows on three-dimensional imaging and spatial precision [14]. Reported outcomes centered on agreement with expert-defined plans and spatial deviation metrics, consistent with outcome measures in digital planning literature [4]. These findings support prior observations that AI currently functions primarily as a decision support tool rather than an autonomous planning system [9].

Prosthodontic applications demonstrated increasing interest in AI-assisted design and digital impression analysis, consistent with trends in CAD/CAM dentistry [2]. Outcomes related to design accuracy and reproducibility align with previous prosthodontic reviews highlighting the potential of automation to enhance workflow consistency [35]. Inclusion of both fixed and removable prosthodontic applications indicates a gradual expansion of AI use within restorative dentistry [6].

From a clinical workflow perspective, most AI applications reviewed remain positioned as adjunctive decision support tools rather than autonomous systems. Diagnostic AI tools were typically evaluated under controlled conditions, and only a limited number of studies assessed their impact on clinician performance, workflow efficiency, or real-time clinical decision-making. Translation into routine practice remains constrained by limited prospective testing, variable workflow integration, and inconsistent reporting of implementation outcomes.

Explainability and interpretability were insufficiently addressed across the included studies. While diagnostic and segmentation models frequently achieved high performance metrics, few studies reported model transparency, feature attribution methods, or clinician-facing explanations that could support trust and safe adoption. This is particularly relevant for implant planning and nerve injury risk assessment, where decision support outputs require clinical justification beyond numerical accuracy.

Regulatory and governance considerations represent additional barriers to clinical translation. Most included studies were retrospective and single-center, limiting generalizability and reducing the level of evidence required for regulatory approval and guideline adoption. Future research would benefit from alignment with regulatory expectations for AI-based medical devices, including external validation, multi-center datasets, reproducibility of model performance, and clear documentation of intended use within digital dental workflows.

Integration of findings across multiple clinical domains provides a structured overview of current AI applications in digital dentistry while acknowledging the need for greater methodological standardization. The limited sample size should therefore be interpreted as reflective of variability in study design and reporting standards rather than inadequate search breadth. Continued efforts toward harmonized validation frameworks and clinically oriented outcome reporting will be essential to support the responsible integration of AI into dental practice.

Limitations and Future Recommendations

This review is limited by heterogeneity in study designs, data modalities, and outcome measures, which restricted direct comparison and precluded quantitative synthesis. Most included studies were based on retrospective datasets and single-center sources, limiting generalizability and reducing comparability across diverse clinical environments. Variation in reporting standards, validation strategies, and reference definitions further complicated evidence integration. The rapid methodological evolution in AI research may also render some findings time-sensitive within a fixed publication window.

Ethical considerations are central to the implementation of AI-supported dental care. Issues related to data privacy, informed consent for secondary use of imaging datasets, and algorithmic bias require careful governance, particularly when models are trained on demographically limited or institution-specific data. Accountability for diagnostic or planning errors involving AI systems remains primarily with the clinician, as current evidence supports AI as a decision support tool rather than an autonomous decision-maker. Clear delineation of responsibility between software developers, institutions, and practitioners is necessary to prevent ambiguity in adverse outcomes.

Clinician oversight remains essential to ensure safe integration of AI outputs into clinical decision-making. AI-generated recommendations should be interpreted within the broader clinical context, including patient history, anatomical variation, and treatment preferences. Over-reliance on automated outputs without critical appraisal may increase the risk of diagnostic anchoring or automation bias. Structured training in AI literacy for dental professionals and transparent model reporting standards would support responsible adoption while maintaining professional judgment as the final determinant of care.

Future research should prioritize prospective, multi-center studies using standardized reporting frameworks to enhance comparability and clinical relevance. Greater emphasis on external validity, workflow integration, explainability, and patient-centered outcomes is required to support real-world implementation. Interdisciplinary collaboration among clinicians, data scientists, ethicists, and regulatory authorities may facilitate the development of robust, accountable, and clinically aligned AI systems. Expansion beyond imaging-dominant tasks toward comprehensive planning and restorative support would further advance the integration of AI into digital dental practice.

## Conclusions

This systematic review summarizes recent research on the use of artificial intelligence in diagnostic assessment, treatment planning, and prosthodontic workflows within digital dentistry. The findings demonstrate that AI has been most extensively applied to image-based diagnostic tasks, where deep learning models show consistent performance in detection, classification, and segmentation outcomes. Treatment planning applications, particularly in implant dentistry, illustrate the role of AI in spatial assessment and risk evaluation. Prosthodontic applications reflect increasing adoption of automation in digital impressions and prosthesis design, with reported improvements in design accuracy and workflow efficiency. Despite heterogeneity in study design and outcome reporting, the evidence indicates progressive integration of AI across interconnected clinical stages.

Current evidence supports AI primarily as a decision support technology embedded within digital workflows rather than as an autonomous replacement for clinician judgment. Reported benefits include enhanced diagnostic consistency, planning precision, and workflow efficiency when AI outputs are interpreted within established clinical reasoning processes. Continued methodological standardization, external validation, and workflow-oriented evaluation will be necessary to support safe and effective clinical translation.
